# Genetic Susceptibility Factors on Genes Involved in the Steroid Hormone Biosynthesis Pathway and Progesterone Receptor for Gastric Cancer Risk

**DOI:** 10.1371/journal.pone.0047603

**Published:** 2012-10-23

**Authors:** Lisa Y. Cho, Jae Jeong Yang, Kwang-Pil Ko, Seung Hyun Ma, Aesun Shin, Bo Youl Choi, Dong Soo Han, Kyu Sang Song, Yong Sung Kim, Soung-Hoon Chang, Hai-Rim Shin, Daehee Kang, Keun-Young Yoo, Sue K. Park

**Affiliations:** 1 Department of Preventive Medicine, Seoul National University College of Medicine, Seoul, Korea; 2 Cancer Research Institute, Seoul National University, Seoul, Korea; 3 Department of Preventive Medicine, Graduate School of Medicine, Gachon University, Incheon, Korea; 4 Molecular Epidemiology Branch, Research Institute, National Cancer Center, Goyang-si, Korea; 5 Department of Preventive Medicine, Hanyang University College of Medicine, Seoul, Korea; 6 Department of Internal Medicine, Hanyang University College of Medicine, Seoul, Korea; 7 Department of Pathology, Chungnam National University College of Medicine, Daejeon, Korea; 8 Medical Genomics Research Center, Korea Research Institute of Bioscience and Biotechnology, Daejeon, Korea; 9 Department of Preventive Medicine, Konkuk University, Chungju, Korea; 10 Cancer Epidemiology Branch, National Cancer Center, Goyang-si, Korea; 11 Non Communicable Diseases and Health Promotion, World Health Organization, Western Pacific Regional Office, Manila, Philippines; 12 Department of Biomedical Science, Seoul National University Graduate School, Seoul, Korea; Baylor College of Medicine, United States of America

## Abstract

**Background:**

The objective of the study was to investigate the role of genes (*HSD3B1*, *CYP17A1*, *CYP19A1*, *HSD17B2*, *HSD17B1*) involved in the steroid hormone biosynthesis pathway and progesterone receptor (*PGR*) in the etiology of gastric cancer in a population-based two-phase genetic association study.

**Methods:**

In the discovery phase, 108 candidate SNPs in the steroid hormone biosynthesis pathway related genes and *PGR* were analyzed in 76 gastric cancer cases and 322 controls in the Korean Multi-Center Cancer Cohort. Statistically significant SNPs identified in the discovery phase were re-evaluated in an extended set of 386 cases and 348 controls. Pooled- and meta-analyses were conducted to summarize the results.

**Results:**

Of the 108 SNPs in steroid hormone biosynthesis pathway related genes and *PGR* analyzed in the discovery phase, 23 SNPs in *PGR* in the recessive model and 10 SNPs in *CYP19A1* in the recessive or additive models were significantly associated with increased gastric cancer risk (*p*<0.05). The minor allele frequencies of the SNPs in both the discovery and extension phases were not statistically different. Pooled- and meta-analyses showed *CYP19A1* rs1004982, rs16964228, and rs1902580 had an increased risk for gastric cancer (pooled OR [95% CI] = 1.22 [1.01–1.48], 1.31 [1.03–1.66], 3.03 [1.12–8.18], respectively). In contrast, all *PGR* SNPs were not statistically significantly associated with gastric cancer risk.

**Conclusions:**

Our findings suggest *CYP19A1* that codes *aromatase* may play an important role in the association of gastric cancer risk and be a genetic marker for gastric cancer susceptibility.

## Introduction

Gastric cancer mortality is the second greatest in the world [1]. Gastric cancer incidence is approximately two times greater among men than women in many regions of the world [2], and the ratio becomes smaller after 60 years of age when most women reach menopause. Gastric cancer incidence in men is more than double than in women in the Korean population (62.2 *vs.* 24.6 per 100,000 persons) [3]. This global consistency of a high male to female incidence ratio in gastric cancer may be due to a hormonal difference between men and women. Thus, it has been hypothesized that female sex steroid hormones, estrogen and progesterone, may play a protective role in gastric cancer incidence.

Although inconsistent, epidemiological studies support this hypothesis. Many epidemiologic studies reported a decreased risk for gastric cancer with greater lifetime exposure to endogenous estrogen [4]–[11], whereas some studies showed no association [12]–[16]. Animal and *in vitro* studies also support this hypothesis. Female and castrated rats had a lower incidence of gastric cancer than non-treated male rats in N-methyl-N’-nitro-N-nitrosoguanidine carcinogenesis model [17]. In *H. pylori*-induced gastric cancer mouse model, 17 beta-estradiol acted as a protective factor in gastric carcinogenesis [18]. Estrogen demonstrated an increase in apoptosis in AGS human gastric cancer cells [19]. In addition, estrogen stimulated expression of trefoil peptides that are important in mucosal protection in the stomach [20]. Although published studies on estrogen influence on gastric cancer risk are inconsistent, a recent meta-analysis supports longer exposure to estrogen effects of either ovarian or exogenous origin may decrease gastric cancer risk [21].

Estrogen and progesterone are synthesized in the steroid hormone biosynthesis pathway. Steroid hormone receptors such as estrogen and progesterone have been identified and are expressed in gastric mucosa and cancer tissues [22]–[26]. Therefore, steroid hormone biosynthesis pathway and their receptors can be altered by genetic variations of related genes, thereby altering and contributing to individual susceptibility to gastric cancer. Of hormonal receptors, in particular, we focused on the progesterone receptor (*PGR*) because progesterone might be a major contributor for gastric carcinogenesis than estrogen. An animal study [27] showed that onapristone, a progesterone anatagonist, inhibited gastric tumor growth as well as estradiol-stimulated growth.

The hypothesis of the current study is genetic polymorphisms involved in the steroid hormone biosynthesis pathway and *PGR* can influence individual susceptibility in the development of gastric cancer. To investigate the hypothesis, a two-phase genetic association study was conducted: 1) the discovery phase was a candidate gene approach analysis focusing on five genes involved in the steroid biosynthesis pathway (*HSD3B1*, *CYP17A1*, *CYP19A1*, *HSD17B2*, and *HSD17B1*) and the hormone receptor gene (*PGR*); 2) the extension phase further examined the most significant SNPs identified in the discovery analysis.

## Materials and Methods

### Study Population

In the discovery phase, the population-based nested case-control study population was recruited from the Korean Multi-Center Cancer Cohort (KMCC), a community-based prospective cohort of participants recruited from four urban and rural areas in Korea (Haman, Chungju, Uljin, and Youngil) from 1993 through 2004 [28]. Participants completed detailed standardized interview based questionnaires on general lifestyle, medical history, physical activity, diet, reproductive factors, pesticide exposure, and additional environmental factors. Blood and spot urine samples were collected and stored at −70°C and −20°C, respectively.

In December 31, 2002, 136 gastric cancer cases in the KMCC were identified through computerized record linkages to the national cancer registry, the national death certificate, and the health insurance medical records. The passive follow-up methods were reported to be 99% efficient and completeness was assured [29]. Cases diagnosed before recruitment (N = 36) and without blood samples (N = 16) were excluded. Cancer-free controls were randomly selected from the KMCC population. There were four controls matched to each gastric cancer case by incidence density sampling based on age (±5 years), sex, residential district, and enrollment. Additionally, eight cases and 14 controls were excluded due to insufficient DNA or poor genotyping. Finally, 76 cases and 322 controls were included in the discovery phase.

In the extension phase, 388 gastric cancer case-control sets were selected as follows. There were 95 new gastric cancer cases and 52 prevalent cases in December 2008 and 52 additional cases whose blood samples were later obtained from the KMCC. In addition, from March 2002 to September 2006, 490 newly diagnosed gastric cancer patients from two university hospitals in Korea that were Chungnam University Hospital and Hanyang University GURI Hospital were identified. Epidemiological data and venous blood samples were collected at time of diagnosis or prior to gastric cancer surgery. Among them, 189 cases with sufficient DNA samples and informed consent were included. Community-based controls matched by age (±5 years), sex, and enrollment year from 2001 to 2005 were randomly selected from the KMCC. There were two cases and 40 controls excluded due to poor genotyping and insufficient sample. Finally, 386 cases and 348 controls were included in the extension phase. Pooled and meta-analyses included 462 cases and 670 controls.

### Ethics Statement

All participants provided written informed consent before entering the studies. The study protocols for the KMCC and current nested case-control studies were approved by the institutional review boards of Seoul National University Hospital and the National Cancer Center of Korea (H-0110-084-002, C-0907-044-2861-170), and Hanyang University Hospital (2003–4).

### Candidate Gene and SNP Selection

There were seven genes in the discovery phase selected from the literature review that were as follows: progesterone receptor (*PGR*); cytochrome P450, family 19, subfamily A, polypeptide 1 (*CYP19A1*); cytochrome P450, family 17, subfamily A, polypeptide 1 (*CYP17A1*); hydroxysteroid (17-beta) dehydrogenase 1 (*HSD17B1*); hydroxysteroid (17-beta) dehydrogenase 2 (*HSD17B2*); hydroxy-delta-5-steroid dehydrogenase, 3 beta- and steroid delta-isomerase 1 (*HSD3B1*).

Candidate single nucleotide polymorphisms (SNP) from selected genes were selected according to the following criteria: 1) reported to have a possible functional significance in previous studies; 2) minor allele frequency (MAF)>0.05 in Asian databases such as SNP500Cancer, HapMap or CGAP using dbSNP IDs (http://www.ncbi.nlm.nih.gov/SNP); 3) concurrently, MAF>0.05 in HapMap Japanese (JET). Finally, 117 SNPS with a design score = 1.1, r^2^>0.8 in five candidate genes in the steroid hormone biosynthesis pathway and *PGR* were genotyped. There were 105 SNPs located in the intron region, eight SNPs located in the promoter region (flanking region or UTR), and four SNPs located in the coding region (Appendix S1).

In the extension phase, SNPs were selected as follows. For *PGR*, in the discovery analysis, 23 SNPs were significant and created one large Haploblock. There were two SNPs of the 23 SNPs located in the coding or 3UTR region. The raw and permutated *p*-values were less than 0.04. For *CYP19A1*, there were ten significant SNPs (raw *p*-value<0.05) located in the intron region. *CYP19A1* created six blocks and the significant SNPs in the discovery phase were located in Blocks 4, 5, and 6.

### Genotyping

Genomic DNA concentrations were measured for all study subjects by a spectrophotometer (NanoDrop ND-1000, NanoDrop Technologies). Genotyping in the discovery phase was performed using GoldenGateTM assay (Illumina®, San Diego, CA). Of the 117 SNPs, nine SNPs were deemed unusable due to failure of genotyping (rs6203 andrs9939740), SNP call rate<90% (rs2236780, rs12594293, rs12592697, rs597255, and rs2830), monomorphism (rs7175531, rs4243229), and were excluded in the analysis. Finally, we analyzed 108 SNPs in six genes (genotyping rate of 99.5%) in 76 cases and 322 controls. To ensure quality control and evaluate intra-subject concordance rate, 52 duplicate samples were randomly distributed in the genotyping plate. Concordance rates for all assays were greater than 99%.

Genotyping in the extension phase was performed using the IlluminaVeraCodeGoldenGate Assay with BeadXpress according to the manufacturer’s protocol (Illumina, San Diego, CA, USA) [16]. To ensure the reliability of the two different genotyping methods, 135 samples (59 cases and 76 controls) were genotyped by both the Genome-Wide Human SNP Array 5.0 and the IlluminaVeraCodeGoldenGate Assay, and the concordance rate was >98.2%. Because of the high concordance rate, all samples were included in the analysis; discordant samples were not eliminated from the analysis.

### Statistical Analysis

Chi-square and Student *t*-test were conducted to compare selected characteristics between gastric cancer cases and controls. Difference in selected characteristics that were sex, age, *H. pylori* infection, CagA and VacAseropositivity, cigarette smoking, alcohol drinking, and gastritis history between cases and controls were determined by a *p*-value of 0.05.

Hardy-Weinberg equilibrium (HWE) was evaluated in the control group for all SNPs using the chi-square test or Fisher’s exact test with a cut-off level of HWE p-value<0.0001. In the discovery analysis, the association between individual SNPs and gastric cancer risk was evaluated based on raw and permutated *p*-values using the likelihood ratio test (LRT) with one degree of freedom in the additive, dominant, and recessive models. The additive model assumes a dose response effect with an increasing number of variant alleles. The dominant and recessive models are tests for the minor allele. If d is the minor allele and D is the major allele, the dominant model is DD *vs.* dd + Dd and the recessive model is dd *vs.* DD + Dd. Permutated *p*-values were estimated by 100,000 permutation tests in the single SNP model. To avoid spurious associations with false positive outcomes, the false discovery rate (FDR) using a Benjamini-Hochberg Method was computed [30]. Gastric cancer risk was calculated as odds ratios (ORs) and 95% confidence intervals (CIs) using unconditional logistic regression model adjusting for risk factors that were age, smoking status (ever *vs.* never), *H. pylori* infection (positive *vs.* negative) and CagA seropositivity (positive *vs.* negative) in the additive, dominant, and recessive models. Haploblocks were created using the default algorithm [31] and tag-SNPs were identified in Haplotype analysis.

In the extension phase, the most significant SNPs in the discovery phase were re-analyzed. Based on the additive and/or recessive models, gastric cancer risk was estimated as OR [95% CI] using unconditional logistic regression model adjusting for the same risk factors as mentioned above. The statistical significance level for the discovery and extension phases was *p*-value<0.05. To summarize the results from the discovery and extension analyses, pooled- and meta-analyses were conducted. Using the fixed effect model, summarized OR [95% CI] were computed. Also, heterogeneity across the studies was evaluated by the Cochran Q statistics [32].

All statistical analyses were performed using SAS software version 9.1 (SAS Institute, Cary, North Carolina), PLINK software version 1.06 (http://pngu.mgh.harvard.edu/purcell/plink) [33], and Haploview 4.1 software (http:www.broadinstitute.org/haploview/haploview).

## Results

There was no significant difference between cases and controls for all selected characteristics in the discovery and extension subjects (*p*>0.05) (Table 1). In the pooled-analysis, a greater number of cases were CagA and VacA seropositive and smokers (*p*<0.05).

**Table 1:** Selected characteristics of gastric cancer cases and controls in the genetic analysis.

	Discovery phase		Extension phase		Total		
	Case(n = 76)N (%)	Control(n = 322)N (%)	Case(n = 386)N (%)	Control(n = 348)N (%)	Case(n = 462)N (%)	Control(n = 670)N (%)	*p*-value
**Age** a	64.5 (8.6)	62.8 (8.4)	61.5 (10.5)	63.1 (8.4)	62.0 (10.3)	63.0 (8.4)	0.10
**Female**	20 (26.3)	98 (30.4)	130 (33.7)	110 (31.6)	150 (32.5)	208 (31.0)	0.61
***H.pylori*** ** infection (+)**	64 (84.2)	271 (84.2)	342 (88.6)	299 (85.9)	406 (87.9)	570 (85.0)	0.18
**CagA (+)**	65 (85.5)	273 (84.8)	355 (92.0)	308 (88.5)	420 (90.9)	581 (86.7)	0.03
**VacA (+)**	44 (57.9)	171 (53.1)	271 (70.2)	233 (67.0)	315 (68.2)	404 (60.6)	<0.01
**Ever smoker** b	52 (68.4)	183 (56.8)	238 (61.7)	191 (54.9)	290 (62.8)	374 (55.8)	0.02
**Ever drinker** c	46 (60.5)	184 (57.1)	239 (62.1)	206 (59.2)	285 (61.8)	390 (58.2)	0.22
**Gastric ulcer history (+)**	8 (10.5)	25 (7.8)	59 (17.6)	50 (18.7)	67 (17.8)	75 (16.8)	0.72

a Mean (SD); median age for total cases and controls was 62.6 years. Age ranged from 29 to 85 years old.

b Ever smokers were defined as former and current smokers.

c Ever drinkers were defined as former and current drinkers.

*P*-value>0.05 for all selected characteristics in the discovery and extension phases.

Of the 108 SNPS in five steroid hormone biosynthesis related genes and *PGR* analyzed in the discovery phase, 23 SNPs in *PGR* in the recessive model and 10 SNPs in *CYP19A1* in the recessive or additive models were significantly associated with increased gastric cancer risk in the single SNP analysis (*p*<0.05). *PGR* rs542384, *PGR* rs543215, *PGR* rs613120, and *PGR* rs1456765 presented 100,000 permutation test *p*<0.01, although FDR p-values were not significant (Table 2).

**Table 2:** Association of significant SNPs in the steroid hormone biosynthesis pathway and *PGR* with gastric cancer risk (discovery phase).

Chr^a^	Gene	db SNP ID	MAF (%)^b^	*p*_global_ c	OR (95% CI)^e^			*p*_permutation_ f
					Dominant	Recessive	Additive	
11	*PGR* d	rs484389^g^	C (18.22)	0.0213	1.21 (0.71–2.07)	3.06 (1.02–9.19)	1.33 (0.86–2.06)	0.0342
		rs500760	G (18.22)	0.0213	1.21 (0.71–2.07)	3.06 (1.02–9.19)	1.34 (0.87–2.07)	0.0342
		rs499699	G (18.26)	0.0217	1.21 (0.71–2.06)	3.05 (1.02–9.16)	1.33 (0.86–2.06)	0.0342
		rs563656	C (18.14)	0.0217	1.22 (0.72–2.08)	3.05 (1.02–9.16)	1.34 (0.87–2.08)	0.0342
		rs523630	T (18.34)	0.0358	1.21 (0.71–2.07)	2.74 (0.93–8.05)	1.32 (0.86–2.04)	0.0477
		rs572402	G (18.22)	0.0213	1.21 (0.71–2.07)	3.06 (1.02–9.19)	1.34 (0.87–2.07)	0.0342
		rs511484	G (18.22)	0.0213	1.21 (0.71–2.07)	3.06 (1.02–9.19)	1.34 (0.87–2.07)	0.0342
		rs526487	T (18.22)	0.0213	1.21 (0.71–2.07)	3.06 (1.02–9.19)	1.34 (0.87–2.07)	0.0342
		rs547378	A (18.26)	0.0363	1.22 (0.72–2.08)	2.74 (0.94–8.02)	1.33 (0.86–2.04)	0.0480
		rs11224575	G (18.39)	0.0197	1.22 (0.71–2.08)	3.12 (1.04–9.36)	1.35 (0.87–2.08)	0.0129
		rs518382	T (18.31)	0.037	1.22 (0.71–2.07)	2.72 (0.93–7.97)	1.32 (0.86–2.04)	0.0477
		rs508533	A (16.08)	0.0118	1.29 (0.75–2.24)	3.99 (1.10–14.46)	1.42 (0.90–2.26)	0.0216
		rs542384	A (15.99)	0.0109	1.23 (0.71–2.14)	4.00 (1.10–14.47)	1.38 (0.86–2.19)	0.0038
		rs491893	A (16.21)	0.0118	1.27 (0.73–2.21)	3.99 (1.10–14.46)	1.41 (0.89–2.24)	0.0216
		rs543215	A (15.91)	0.0111	1.24 (0.71–2.16)	3.98 (1.10–14.42)	1.38 (0.87–2.21)	0.0074
		rs613120	C (15.91)	0.0111	1.24 (0.71–2.16)	3.98 (1.10–14.42)	1.38 (0.87–2.21)	0.0074
		rs1456764^g^	A (15.70)	0.0118	1.36 (0.78–2.35)	3.92 (1.08–14.26)	1.47 (0.93–2.33)	0.0216
		rs1456765	T (15.74)	0.0109	1.38 (0.80–2.40)	4.08 (1.13–14.77)	1.50 (0.94–2.38)	0.0032
		rs7106686	A (15.83)	0.0118	1.33 (0.77–2.31)	3.99 (1.10–14.46)	1.46 (0.92–2.31)	0.0216
		rs566351	T (16.25)	0.0245	1.38 (0.80–2.38)	3.31 (0.96–11.38)	1.46 (0.93–2.31)	0.0389
		rs537681	T (16.25)	0.0245	1.39 (0.80–2.39)	3.31 (0.96–11.36)	1.47 (0.93–2.31)	0.0389
		rs501732	T (18.09)	0.0241	1.17 (0.69–2.01)	3.32 (0.97–11.41)	1.30 (0.83–2.05)	0.0389
		rs529359	A (16.16)	0.0228	1.32 (0.76–2.29)	3.31 (0.96–11.36)	1.42 (0.90–2.24)	0.0178
15	*CYP19A1*	rs16964228^g^	T (13.10)	0.0511	1.68 (0.96–2.93)	2.95 (0.67–12.94)	1.64 (1.01–2.66)	0.0488
		rs1902580^g^	A (16.79)	0.0328^d^	1.05 (0.61–1.81)	4.84 (1.15–20.33)	1.25 (0.77–2.02)	0.0351
		rs936306^g^	T (31.06)	0.0341	1.65 (0.97–2.78)	1.81 (0.79–4.12)	1.55 (1.04–2.30)	0.0359
		rs2470176	G (31.68)	0.0295	1.62 (0.96–2.74)	1.86 (0.84–4.12)	1.52 (1.03–2.24)	0.0300
		rs16964254	G (30.81)	0.0248	1.62 (0.96–2.73)	2 (0.90–4.43)	1.54 (1.04–2.27)	0.0249
		rs8031463	C (30.93)	0.0333	1.58 (0.94–2.66)	1.95 (0.88–4.32)	1.51 (1.02–2.22)	0.0348
		rs10519301	A (17.05)	0.0119^d^	1.10 (0.64–1.88)	5.64 (1.47–21.7)	1.30 (0.81–2.08)	0.2244
		rs1004982^g^	G (29.27)	0.0372	1.56 (0.94–2.61)	1.91 (0.87–4.22)	1.51 (1.03–2.21)	0.0396
		rs7168331	C (29.27)	0.0372	1.56 (0.94–2.60)	1.91 (0.87–4.22)	1.48 (1.01–2.17)	0.0401
		rs1870049	C (18.34)	0.0619	11.64 (0.98–2.75)	1.68 (0.51–5.49)	1.51 (0.99–2.32)	0.0605

a Chromosome.

b Minor allele frequency.

c All raw *p*-values calculated with 1 degree of freedom in additive model except rs1902580, rs10519301.

d Rs1902580, rs10519301 raw *p*-values calculated with 1 degree of freedom in recessive model.

e Adjusted for age, smoking, history of *H. pylori* infection, and CagA infection.

f Permutated *p*-values calculated from 10,000 permutations in the single SNP analysis in the additive model.

g SNPs selected for the extension analysis. For *PGR,* one haploblock created and thus SNP selected according to the following criteria: 1) SNPs on coding or 3UTR region; 2) lower raw p-value and permutated *p*-value<0.04; 3) tag-SNP using tagger in Haploview. For *CYP19A1,* six haploblocks created and significant SNPs in discovery phase were located in the blocks 4, 5, and 6. SNP selection for the extension phase was one or more of the following: 1) lower raw *p*-value and permutated *p*-value<0.04; 2) tag-SNP using tagger in Haploview.

h All FDR *p*-values>0.05.

**Figure 1:**
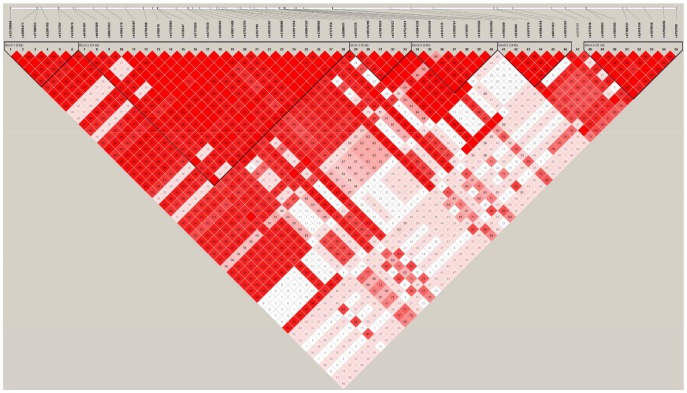
*CYP19A1* gene map and LD block. D’ and LOD values were used for selection of LD color scheme in the discovery phase. Of the six blocks in *CYP19A1*, significant SNPs in the discovery phase were located in blocks 4, 5, and 6.

**Figure 2:**
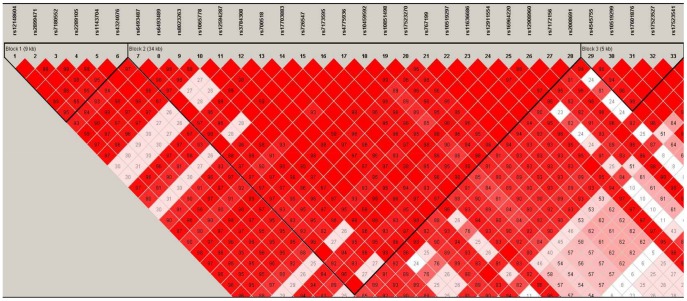
*CYP19A1* gene map blocks 1, 2, and 3.

**Figure 3:**
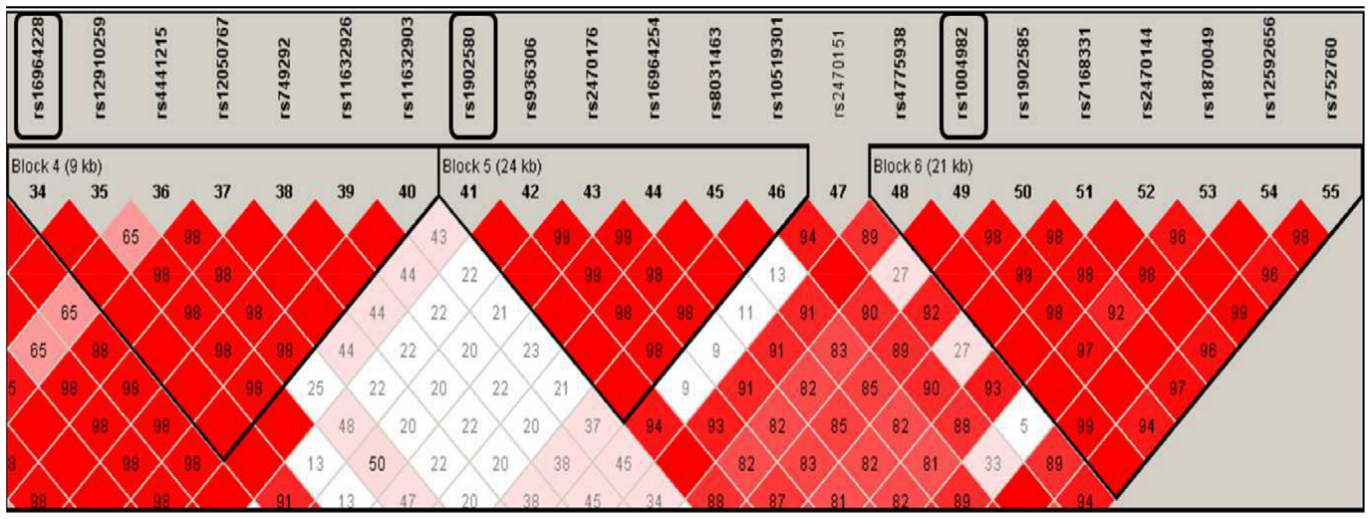
*CYP19A1* gene map blocks 4, 5, and 6. Significant SNPs in the discovery phase were located in blocks 4, 5, and 6. SNPs indicated in boxes represent SNPs re-analyzed in the extension phase.

Haplotype blocks were identified by the LD plot. One block was defined by *PGR* that included all 27 *PGR* SNPs from the discovery phase (Figure S1), while six blocks were defined by *CYP19A1* (Figures 1,2, and 3).

The minor allele frequencies of the SNPs in both the discovery and extension phases were not statistically different. Pooled- and meta-analyses showed *CYP19A1* was statistically significantly associated with gastric cancer risk. Minor alleles G, T, and A for rs1004982, rs16964228, and rs1902580, respectively, reported a 1.22 (95% CI [1.01–1.48]), 1.31 (95% CI [1.03–1.66]), and 3.03 (95% CI [1.12–8.18]) increased risk for gastric cancer, respectively, in the pooled-analysis. Meta-analysis showed similar associations. In contrast, all *PGR* SNPs were not statistically significantly associated with gastric cancer risk (Table 3).

**Table 3:** Association of most significant SNPs in the steroid hormone biosynthesis pathway with gastric cancer risk in the pooled- and meta-analyses.

Gene	Chr^a^	SNP	Minorallele	MAF^b^			Genetic model	OR (95% CI)	
				Discovery	Extension	Pooled		Pooled-analysis^c^	Meta-analysis^d^
***CYP19A1***	15	rs1004982	G	0.2764	0.2796	0.2780	Additive	**1.22 (1.01–1.48)**	**1.25 (1.02–1.52)**
							Dominant	**1.27 (1.00–1.61)**	1.25 (0.97–1.62)
							Recessive	1.40 (0.91–2.17)	1.53 (0.97–2.42)
		rs16964228	T	0.1196	0.1371	0.1287	Additive	**1.31 (1.03–1.66)**	1.27 (0.99–1.63)
							Dominant	**1.34 (1.03–1.75)**	1.27 (0.96–1.68)
							Recessive	1.57 (0.72––3.45)	1.61 (0.72–3.62)
		rs1902580	A	0.1621	0.1653	0.1636	Additive	**1.27 (1.00–1.61)**	1.25 (0.97–1.62)
							Dominant	0.93 (0.72–1.20)	0.91 (0.70–1.19)
							Recessive	**3.03 (1.12–8.18)**	**4.10 (1.43–11.8)**

a Chromosome.

b Minor allele frequency.

c Adjusted for age, smoking, history of *H. pylori* infection, and CagA infection.

d No heterogeneity (Cochran Q test, *P*-heterogeneity>0.05) except rs1456764 (*p* = 0.027) in the recessive mode.

## Discussion

*CYP19A1* genetic polymorphisms, specifically rs1004982, rs16964228, rs1902580, were associated with an increased risk for gastric cancer in the current study. The discovery analysis showed 23 SNPs in *PGR* were associated with increased gastric cancer risk and created one large haploblock in haplotype analysis, although associations were not significant in the pooled-analysis.

*CYP19A1* encodes *CYP19* aromatase, a member of the cytochrome P450 superfamily that is the main enzyme that catalyzes the final and rate-limiting step of estrogen biosynthesis (aromatization of androstenedione and testosterone to estrone and estradiol, respectively) [34]. *CYP19* gene is mapped to chromosome 15q21.1, spans about 123 kb, and the regulatory region contains at least 190 distinct promoters that regulate in a signal pathway-specific manner [35] or tissue-specific with hormonally controlled promoters such as gonadal or adipose stroma [36]–[39]. *CYP19* mutations have demonstrated increased or decreased aromatase activity thereby altering levels of circulating estrogen [40]–[43]. *CYP19A1* genetic variation related studies investigated the association with various hormone related cancers such as breast, endometrial, ovarian, and prostate [44]–[49]. Aromatase activity stimulated breast cancer cell growth [50], aromatase expression levels increased in breast tumors [51], and was the main source of 17β-estradiol in breast tumors and surrounding tissues in postmenopausal women [52], [53]. Studies on the role of *CYP19A1* specific to human gastric carcinogenesis are limited. However, strong expression of mRNA *CYP19* aromatase was shown in gastric mucosa in adult rats, and aromatase activity in gastric carcinoma human specimens was demonstrated [25]. This suggests a mechanism that polym encoding orphic variants of *CYP19* genes may affect cancer susceptibility by altering its encoded enzyme, either through expression or function, to modulate estrogen synthesis. Our findings suggest the possibility that genetic variants of *CYP19A1* (rs1004982, rs16964228, and rs1902580) might be involved in altering estrogen levels and affecting apoptosis, mucosal function, carcinogenesis, and thus gastric cancer risk.

There are limited studies that examine genes of the steroid hormone metabolism pathway and gastric cancer. A Japanese study observed a statistically significant association between several *CYP19A1* SNPs (rs4646 and rs1902586) and gastric cancer risk [54]. A population-based study in Poland that included 295 gastric cancer cases and 415 controls also genotyped a couple of the same SNPs (rs4646 and rs1902586), however, a significant association was not found [55]. These SNPs were not genotyped in our study, however, other SNPs of *CYP19A1* showed statistically significant associations. We genotyped *CYP19A1* rs16964228, rs1902580, and rs1004982 that are located in the intron region in three blocks, Block 4, Block 5, and Block 6, respectively. Although the functional relevance of *CYP19A1* rs16964228, rs1902580, and rs1004982 for *CYP19* enzyme is not clear, *CYP19A1* may act as a key marker of individual susceptibility and its genetic variants can modify the development of gastric cancer, but further confirmation is warranted.

Many studies examined *CYP19A1* with hormonally associated cancers, such as breast, prostate, endometrial [56]–[59]. SNPs rs10046 (T) and rs936306 (T) are suggested to be ‘high activity alleles’ due to their association with 10% to 20% increased levels of circulating estradiol and estrone in postmenopausal women [58], [60], [61], although did not show significant association with breast cancer [60], [62]. In the current study, rs10046 was not genotyped, but rs936306 was genotyped. While rs936306 was significant in the discovery phase, rs936306 was insignificant in the pooled and meta-analyses.

Our discovery analysis showed 23 SNPs in *PGR* were associated with an increased risk for gastric cancer. In our haplotype analysis, the significant 23 SNPs from the discovery analysis, in addition to the remaining four *PGR* SNPs that were genotyped, formed one large block, suggesting these SNPs are correlated with each other and are associated with gastric cancer. However, due to insufficient power in the recessive model, the extension phase did not report a statistically significant association with any *PGR* SNPs. *PGR* levels were significantly increased in gastric cancer patients’ tissues while not in normal tissue [63] suggesting gastric mucosa may be the target tissue for progesterone action [64]. Therefore, polymorphic variants of *PGR* may be involved in modification of gastric cancer susceptibility by altering its encoded receptor status expression and function.

Although this was a two phase study that aimed to increase the number of study subjects, the power was nevertheless low, and did not allow stratified analysis according to hormone related factors such as menopausal status, gender, and cancer type such as cardiac and non-cardiac. The etiology of gastric cancer is multi-factorial, and an in-depth understanding of risk and protective factors and its interactions will help provide an even better understanding of the disease. Moreover, in the extension phase, hospital and community-based cases were matched to community-based controls that may introduce bias. However, information bias was minimized since people are born with their genes and changes in genes are not common. Also, selection bias was minimized because cases were matched to controls according to important risk factors in the initial study design stage.

The study is a two-phase genetic association study. In the candidate approach genetic analysis, significant SNPs that were identified in the discovery phase were re-analyzed in the extension phase. Second, this population-based nested case-control study is free of many biases common in retrospective designs. Confounding factors were adjusted for in multivariate models.

In summary, this population-based two-phase genetic association study reports *CYP19A1* genetic variants, rs16964228, rs1902580, and rs1004982, are significantly associated with gastric cancer risk and appear to be a genetic marker of susceptibility in gastric carcinogenesis in the Korean population. Given *CYP19A1*’s key role in estrogen biosynthesis, *CYP19A1* polymorphisms that alter estrogen production can be involved in gastric carcinogenesis. Future studies of estrogen and testosterone biomarkers from blood and urine are needed to confirm and further understand the molecular basis.

## Supporting Information

Figure S1 ***PGR*** **gene map and LD block.** D’ and LOD values were used for selection of LD color scheme in the discovery phase. SNPs indicated in boxes represent SNPs re-analyzed in the extension.
(TIFF)

Appendix S1 **Detailed information on the candidate genes and SNPs in the steroid hormone biosynthesis pathway and** ***PGR*** **.**
(DOCX)
