# Supplementary figures and images for: Genetic Susceptibility Factors on Genes Involved in the Steroid Hormone Biosynthesis Pathway and Progesterone Receptor for Gastric Cancer Risk
Source: PLoS One. 2012 Oct 23;7(10):e47603. doi: 10.1371/journal.pone.0047603 (PMC3479131; doi:10.1371/journal.pone.0047603)

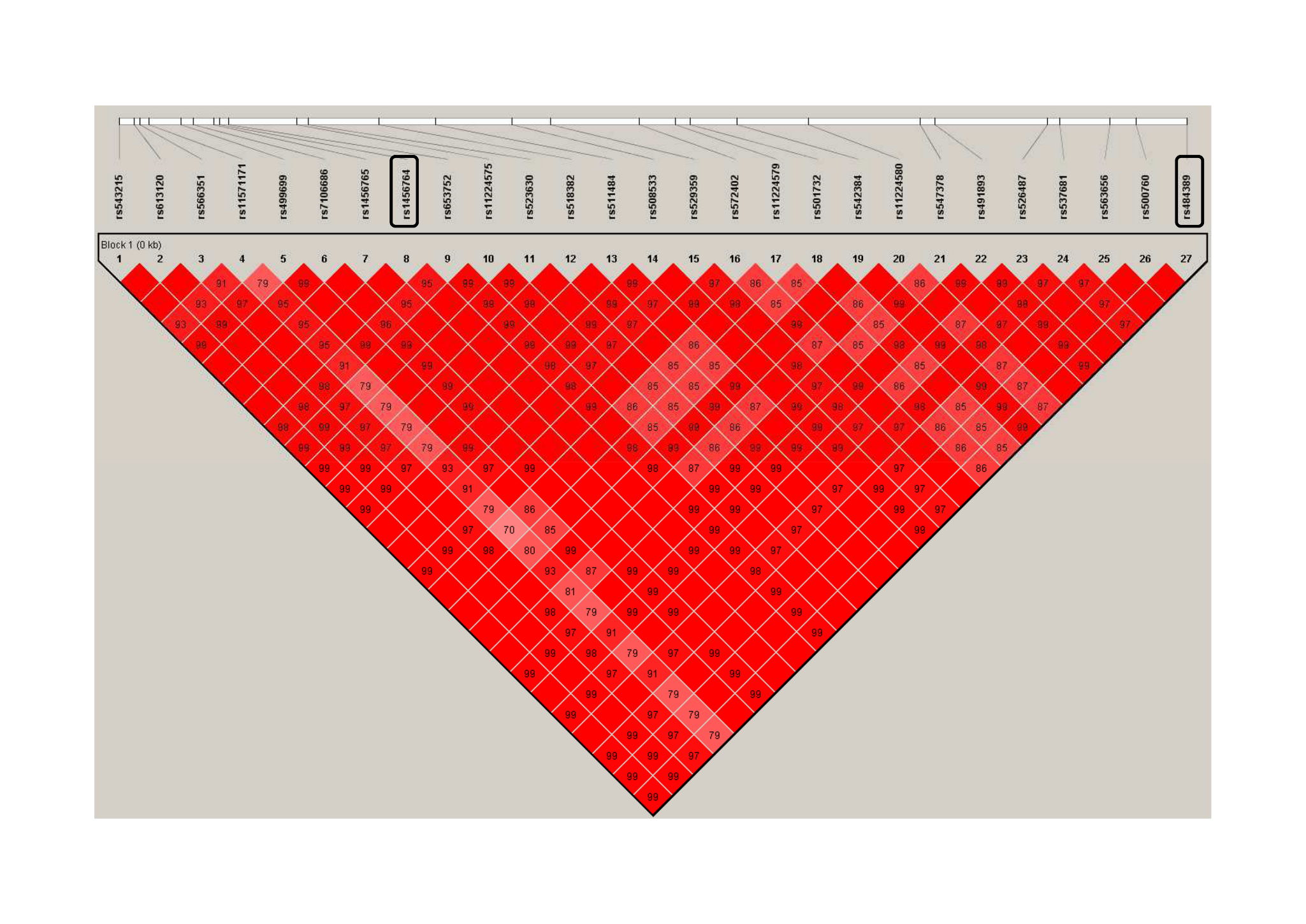

Supplement: Figure S1 — PGR gene map and LD block. D’ and LOD values were used for selection of LD color scheme in the discovery phase. SNPs indicated in boxes represent SNPs re-analyzed in the extension. (TIFF) [file pone.0047603.s001.tiff]
