# Supplementary material for: Genetic Susceptibility Factors on Genes Involved in the Steroid Hormone Biosynthesis Pathway and Progesterone Receptor for Gastric Cancer Risk
Source: PLoS One. 2012 Oct 23;7(10):e47603. doi: 10.1371/journal.pone.0047603 (PMC3479131; doi:10.1371/journal.pone.0047603)
Supplement: Appendix S1 — Detailed information on the candidate genes and SNPs in the steroid hormone biosynthesis pathway and PGR . (DOCX) [file pone.0047603.s002.docx]

**Appendix S1. Detailed information on the candidate genes and SNPs in the steroid hormone biosynthesis pathway and *PGR***

| **Chr^a^** | **Gene** | **Gene description** | **db SNP ID** | **Chr position** | **Nucleotide (protein) change** | **MAF (%)^b^** | **HWE^c^** | **Genotyping^d^** |
| --- | --- | --- | --- | --- | --- | --- | --- | --- |
| 1 | *HSD3B1* | Hydroxy-delta-5-steroid dehydrogenase, 3 beta- and steroid delta-isomerase 1 | rs6203 | 11985868 | Coding [701/110] T>C | 0 | - | 0.00 |
|  |  |  | rs3765945 | 119852969 | Intron -1202 T>C | C (6.19) | 0.8194 | 100.00 |
|  |  |  | rs10754400 | 119859505 | Flanking_3UTR -308 T>G | G (6.37) | 0.7755 | 99.77 |
|  |  |  | rs2236780 | 119851986 | Intron -219 A>G | A (6.21) | 0.9059 | 85.48 |
| 10 | *CYP17A1* | Cytochrome P450, family 17, subfamily A, polypeptide 1 | rs10786712 | 104586386 | Intron -426 T>C | T (49.20) | 0.0400 | 96.77 |
|  |  |  | rs3781287 | 104585410 | Intron -271 A>C | A (49.84) | 0.0200 | 97.70 |
|  |  |  | rs2486758 | 104587470 | Flanking_5UTR -190 T>C | C (24.92) | 0.7800 | 100.00 |
|  |  |  | rs3740397 | 104582665 | Intron -75 C>G | G (39.22) | 0.1800 | 98.62 |
|  |  |  | rs1004467 | 104584497 | Intron -35 T>C | C (29.57) | 0.1200 | 100.00 |
|  |  |  | rs17115100 | 104581383 | Intron -25 T>G | T (30.80) | 0.0300 | 100.00 |
|  | *PGR* | Progesterone receptor | rs484389 | 100415019 | 3UTR [706/37] T>C | C (17.65) | 0.4300 | 100.00 |
|  |  |  | rs500760 | 100415201 | Coding [144/11] A>G | C (17.65) | 0.4300 | 100.00 |
|  |  |  | rs1456764 | 100487654 | Coding [144/11] A>T | A (15.02) | 0.3200 | 100.00 |
|  |  |  | rs1456765 | 100487782 | Intron -14166 T>G | T (15.02) | 0.3200 | 99.77 |
|  |  |  | rs7106686 | 100488308 | Intron -13640 A>G | A (15.17) | 0.2900 | 100.00 |
|  |  |  | rs566351 | 100490224 | Intron -11724 T>C | T (15.53) | 0.4500 | 99.54 |
|  |  |  | rs613120 | 100479488 | Intron -11671 T>C | C (15.37) | 0.2600 | 99.08 |
|  |  |  | rs543215 | 100479243 | Intron -11426 A>G | A (15.37) | 0.2600 | 99.08 |
|  |  |  | rs537681 | 100493244 | Intron -8704 T>C | T (15.53) | 0.4500 | 99.77 |
|  |  |  | rs491893 | 100474357 | Intron -6540 A>G | A (15.63) | 0.2200 | 100.00 |
|  |  |  | rs542384 | 100471848 | Intron -4031 A>T | A (15.48) | 0.2400 | 99.54 |
|  |  |  | rs501732 | 100498282 | Intron -3666 T>C | T (17.49) | 0.1300 | 100.00 |
|  |  |  | rs572402 | 100422465 | Intron -3405 A>G | C (17.65) | 0.4300 | 100.00 |
|  |  |  | rs529359 | 100499056 | Intron -2892 A>G | A (15.53) | 0.4500 | 99.31 |
|  |  |  | rs508533 | 100470449 | Intron -2632 A>C | A (15.48) | 0.2400 | 100.00 |
|  |  |  | rs511484 | 100423643 | Intron -2227 C>G | C (17.65) | 0.4300 | 100.00 |
|  |  |  | rs518382 | 100436327 | Intron -2061 T>C | T (17.76) | 0.6700 | 99.54 |
|  |  |  | rs523630 | 100419857 | Intron -1814 T>C | T (17.80) | 0.6400 | 100.00 |
|  |  |  | rs11224575 | 100429243 | Intron -1734 A>G | G (17.80) | 0.4000 | 99.77 |
|  |  |  | rs563656 | 100416205 | Intron -993 T>C | C (17.55) | 0.4600 | 99.54 |
|  |  |  | rs547378 | 100428149 | Intron -640 A>G | A (17.70) | 0.6800 | 99.77 |
|  |  |  | rs499699 | 100415342 | Intron -130 A>G | G (17.70) | 0.4200 | 99.77 |
|  |  |  | rs526487 | 100425785 | Intron -85 A>T | T (17.65) | 0.4300 | 100.00 |
|  |  |  | rs653752 | 100453320 | Intron -14831 C>G | G (15.17) | 0.2900 | 100.00 |
|  |  |  | rs11571171 | 100480097 | Intron -12280 T>C | C (19.81) | 0.6400 | 100.00 |
|  |  |  | rs11224580 | 100443503 | Intron -4810 T.>C | C (18.11) | 0.5500 | 100.00 |
|  |  |  | rs11224579 | 100442270 | Intron -3577 T>C | C (22.45) | 0.9300 | 100.00 |
| 15 | *CYP19A1* | Cytochrome P450, family 19, subfamily A, polypeptide 1 | rs1902580 | 49362793 | Intron -40354 A>G | A (16.30) | 0.0600 | 99.31 |
|  |  |  | rs936306 | 49366890 | Intron -36415 T>C | T (29.50) | 0.1800 | 99.31 |
|  |  |  | rs2470176 | 49371231 | Intron -32074 A>G | G (30.03) | 0.2100 | 98.85 |
|  |  |  | rs16964254 | 49383073 | Intron -20232 T>G | G (29.13) | 0.2500 | 99.53 |
|  |  |  | rs16964228 | 49342247 | Intron -19808 T>C | T (11.92) | 0.8300 | 99.77 |
|  |  |  | rs8031463 | 49383831 | Intron -19474 T>C | C (29.35) | 0.3200 | 99.31 |
|  |  |  | rs10519301 | 49386829 | Intron -16476 A>G | A (16.36) | 0.0600 | 99.54 |
|  |  |  | rs700518 | 49316404 | Coding [56/94] A>G | G (44.72) | 0.4200 | 99.54 |
|  |  |  | rs11632903 | 49351633 | Intron -29194 T>C | T (46.42) | 0.7900 | 99.54 |
|  |  |  | rs11632926 | 49351518 | Intron -29079 A>G | A (46.58) | 0.8400 | 99.77 |
|  |  |  | rs749292 | 49346023 | Intron -23954 A>G | A (46.57) | 0.8900 | 99.08 |
|  |  |  | rs12050767 | 49344549 | Intron -22110 T>C | C (46.58) | 0.8400 | 99.77 |
|  |  |  | rs4441215 | 49344251 | Intron -21812 C>G | G (41.49) | 0.1300 | 100.00 |
|  |  |  | rs12910259 | 49342341 | Intron -19902 A>G | G (46.88) | 0.5500 | 100.00 |
|  |  |  | rs17523541 | 49342136 | Intron -19697 A>G | G (46.72) | 0.4800 | 98.39 |
|  |  |  | rs17523527 | 49341324 | Intron -18885 T>C | G (46.75) | 0.5900 | 200.00 |
|  |  |  | rs17601876 | 49341201 | Intron -18762 A>G | A (35.14) | 0.6500 | 100.00 |
|  |  |  | rs10519299 | 49338638 | Intron -16199 C>G | C (46.58) | 0.4800 | 99.77 |
|  |  |  | rs4545755 | 49336336 | Intron -13897 A>G | A (44.10) | 0.5900 | 99.54 |
|  |  |  | rs2008691 | 49335602 | Intron -13163 A>G | G (22.60) | 0.4300 | 100.00 |
|  |  |  | rs7172156 | 49333590 | Intron -11151 A>G | A (32.97) | 0.7800 | 99.54 |
|  |  |  | rs12908960 | 49333152 | Intron -10713 A>G | A (44.24) | 0.4700 | 99.31 |
|  |  |  | rs2470151 | 49394361 | Intron -8944 A>G | A (43.40) | 0.0300 | 98.39 |
|  |  |  | rs16964220 | 49330674 | Intron -8235 A>G | A (22.60) | 0.6300 | 100.00 |
|  |  |  | rs12911554 | 49330049 | Intron 7610 T>C | C (33.44) | 0.7800 | 99.77 |
|  |  |  | rs11636686 | 49329358 | Intron -6919 A>G | A (43.61) | 0.5000 | 99.08 |
|  |  |  | rs10519297 | 49328952 | Intron -6513 A>G | A (43.81) | 0.5000 | 100.00 |
|  |  |  | rs4775938 | 49397067 | Intron -6238 C>G | G (31.11) | 0.1400 | 100.00 |
|  |  |  | rs2470144 | 49409017 | Intron -5604 A>G | A (36.69) | 0.4100 | 100.00 |
|  |  |  | rs1870049 | 49412515 | Intron -5469 T>C | C (17.03) | 0.5200 | 100.00 |
|  |  |  | rs767199 | 49327679 | Intron -5240 A>G | A (43.81) | 0.5000 | 100.00 |
|  |  |  | rs7168331 | 49408275 | Intron -4862 C>G | C (27.71) | 0.8200 | 100.00 |
|  |  |  | rs12594293 | 49311349 | Intron -3927 C>G | G (22.07) | 0.5200 | 85.48 |
|  |  |  | rs12592697 | 49312465 | Intron -3883 T>C | T (32.07) | 0.6200 | 85.48 |
|  |  |  | rs12594287 | 49311199 | Intron -3777 A>G | A (22.60) | 0.8700 | 99.77 |
|  |  |  | rs17523270 | 49325572 | Intron -3133 T>G | T (42.11) | 0.3900 | 100.00 |
|  |  |  | rs8023263 | 49304889 | Intron -2379 T>G | G (45.96) | 0.3700 | 99.54 |
|  |  |  | rs1004982 | 49401103 | Intron -2202 A>G | G (27.71) | 0.8200 | 100.00 |
|  |  |  | rs1902585 | 49401198 | Intron -2107 C>G | C (37.07) | 0.4600 | 98.95 |
|  |  |  | rs10851498 | 49324304 | Intron -1865 T>C | C (41.77) | 0.3800 | 99.54 |
|  |  |  | rs12592656 | 49416238 | Intron -1746 T>G | T (30.69) | 0.1300 | 98.85 |
|  |  |  | rs3784308 | 49314690 | Intron -1658 A>G | G (22.74) | 0.9000 | 99.08 |
|  |  |  | rs7173595 | 49321028 | Intron -1229 T>C | C (31.21) | 0.5400 | 99.77 |
|  |  |  | rs10459592 | 49323433 | Intron -994 T>G | G (43.48) | 0.9800 | 99.54 |
|  |  |  | rs726547 | 49317459 | Intron -961 T>C | T (22.60) | 0.8700 | 100.00 |
|  |  |  | rs7175531 | 49321347 | Intron -910 T>C | T (00.00)) | - | 90.32 |
|  |  |  | rs17703883 | 49317389 | Intron -891 T>C | C (30.90) | 0.6500 | 99.77 |
|  |  |  | rs4775936 | 49323314 | Intron -875 T>C | T (41.93) | 0.4400 | 99.54 |
|  |  |  | rs6493487 | 49301021 | Intron -817 A>G | G (29.72) | 0.6800 | 100.00 |
|  |  |  | rs2899471 | 49293845 | Intron -714 A>G | G (43.93) | 0.8100 | 99.31 |
|  |  |  | rs752760 | 49418771 | Flanking_5UTR -685 T>C | G (37.58) | 0.7300 | 99.77 |
|  |  |  | rs7180552 | 49294060 | Intron -499 T>G | T (43.48) | 0.8000 | 99.31 |
|  |  |  | rs6493489 | 49301495 | Intron -343 T>C | C (44.84) | 0.5500 | 98.85 |
|  |  |  | rs12148604 | 49288696 | Flanking_3UTR -265 T>C | C (43.04) | 0.8500 | 100.00 |
|  |  |  | rs2289105 | 49294800 | Intron -79 T>C | T (43.50) | 0.8400 | 100.00 |
|  |  |  | rs1065778 | 49307498 | Intron -76 A>G | G (44.74) | 0.6000 | 100.00 |
|  |  |  | rs1143704 | 49297994 | Intron -36 A>T | A (44.43) | 0.6100 | 100.00 |
|  |  |  | rs4324076 | 49298160 | Intron -16 A>C | A (43.95) | 0.7300 | 99.77 |
| 16 | *HSD17B2* | Hydroxysteroid (17-beta) dehydrogenase 2 | rs4243229 | 80643099 | Intron -16177 A>G | G (00.00) | - | 99.78 |
|  |  |  | rs11860188 | 80642931 | Intron -16136 A>T | A (25.00) | 0.0193 | 99.54 |
|  |  |  | rs9788807 | 80675563 | Intron -6445 A>C | C (39.63) | 0.6884 | 100.00 |
|  |  |  | rs11150437 | 80653371 | Intron -5905 T>C | C (25.70) | 0.8448 | 100.00 |
|  |  |  | rs1559427 | 80677171 | Intron -4837 T>C | C (39.78) | 0.7945 | 99.54 |
|  |  |  | rs4889452 | 80631174 | Intron -4379 A>G | G (25.93) | 0.4415 | 99.54 |
|  |  |  | rs2955163 | 80685698 | Intron -3483 C>G | G (32.97) | 0.4328 | 100.00 |
|  |  |  | rs8059915 | 80630245 | Intron -3450 C>G | G (25.08) | 0.2748 | 99.54 |
|  |  |  | rs1364285 | 80685844 | Intron -3337 C>G | G (36.38) | 0.6750 | 100.00 |
|  |  |  | rs4888202 | 80685923 | Intron -3258 T>C | T (38.54) | 0.9978 | 100.00 |
|  |  |  | rs2966246 | 80686528 | Intron -2653 C>G | C (27.40) | 0.0805 | 100.00 |
|  |  |  | rs9939740 | 80679482 | Intron -2526 A>G | 0 | - | 0.00 |
|  |  |  | rs2966248 | 80684417 | Intron -2272 T>G | T (19.97) | 0.0747 | 100.00 |
|  |  |  | rs9940004 | 80686986 | Intron -2195 T>C | T (36.38) | 0.6750 | 100.00 |
|  |  |  | rs9934209 | 80684331 | Intron -2186 C>G | C (38.63) | 0.7957 | 99.08 |
|  |  |  | rs6564964 | 80684225 | Intron -2080 T>G | G (35.29) | 0.7634 | 100.00 |
|  |  |  | rs996752 | 80690493 | Flanking_3UTR -855 T>C | C (23.62) | 0.8243 | 100.00 |
|  |  |  | rs8191138 | 80659790 | Intron -302 A>G | A (24.30) | 0.0363 | 100.00 |
| 17 | *HSD17B1* | Hydroxysteroid (17-beta) dehydrogenase 1 | rs592389 | 37960970 | Flanking_3UTR -213 T>G | G (40.37) | 0.9107 | 99.77 |
|  |  |  | rs676387 | 37959799 | Intron -150 T>G | T (38.98) | 0.9839 | 98.85 |
|  |  |  | rs597255 | 37958626 | Intron -42 T>C | T (39.45) | 0.9939 | 85.25 |
|  |  |  | rs2830 | 37958089 | 5UTR [579/388] A>G | A (39.14) | 0.8866 | 85.48 |
